# Ex Vivo Model to Assess the Exposure of Patients to Plasticizers from Medical Devices during Pre-CAR-T Cells’ Apheresis

**DOI:** 10.3390/toxics10020079

**Published:** 2022-02-08

**Authors:** Raphaëlle Lautraite, Lise Bernard, Pascale Halle, Philip Chennell, Yoann Le Basle, Justyna Kanold, Valérie Sautou

**Affiliations:** 1 Université Clermont Auvergne, CHU Clermont Ferrand, Clermont Auvergne INP, CNRS, ICCF, F-63000 Clermont-Ferrand, France; rlautraite@hotmail.fr (R.L.); pchennell@chu-clermontferrand.fr (P.C.); ylebasle@chu-clermontferrand.fr (Y.L.B.); vsautou@chu-clermontferrand.fr (V.S.); 2 CHU Clermont-Ferrand, Centre de Biothérapie d’Auvergne, F-63000 Clermont-Ferrand, France; phalle@chu-clermontferrand.fr (P.H.); jkanold@chu-clermontferrand.fr (J.K.); 3 Université Clermont Auvergne, CHU Clermont-Ferrand, INSERM CIC 1405 Unité CRECHE, F-63000 Clermont–Ferrand, France

**Keywords:** exposure, leukapheresis, CAR-T cells, medical devices, plasticizer, di(2-ethylhexyl) phthalate

## Abstract

Background: The treatment of relapsed or refractory leukemia remains a major problem. Among the new therapeutic approaches, the use of modified T lymphocytes, called chimeric antigen receptor T cells (CAR-T cells), seems promising. The first step of their preparation is leukapheresis, which involves the collection of mononuclear cells from the patient. This medical procedure requires numerous medical devices (MDs) made of plasticized polyvinylchloride (PVC). These compounds can leach out of the devices during contact with the patient’s blood. The aim of our study was to evaluate the migration of the plasticizers contained in the MD during a simulated pre-CAR-T cell leukapheresis procedure, and to measure the patient’s and their lymphocytes’ exposure to them. Methods: The qualitative and quantitative composition of the MD used for pre-CAR-T cell apheresis was determined by gas chromatography–mass spectrometry (GC–MS). Then, an ex vivo leukapheresis model using an ethanol/water simulant was performed to evaluate the plasticizers’ migration under simulated clinical conditions of pre-CAR-T cells’ cytapheresis. The plasticizers released into the simulant were quantified by GC–MS. Results: Diethylhexylphthalate (DEHP) was found in the apheresis kit, with amounts ranging from 25% to 59% (g/100 g of PVC). Bis(2-ethylhexyl) adipate was detected at trace levels. A total of 98.90 ± 11.42 mg of DEHP was released into the simulant, corresponding to an exposure dose of 1.4 mg/kg for a 70 kg patient. Conclusions: Patients undergoing a pre-CAR-T cell apheresis are mainly exposed to DEHP, which can impact their health because of its endocrine disruption effect, but could also lead to a decrease in CAR-T cells’ efficiency/quality.

## 1. Introduction

Each year, refractory lymphoid leukemia and lymphoma cause many deaths worldwide—35,000 and 269,000, respectively [1]. Recently, the expansion of immunotherapy has led to the development of new innovative therapies such as modified T lymphocytes, called chimeric antigen receptor T cells (CAR-T cells). CAR-T cells’ immunotherapy is composed of autologous T= lymphocytes taken from the patient and genetically modified ex vivo to express the CAR receptor.

These treatments are an important step forwards for the management of DLBCL patients, and some newly marketed drugs have already proven their efficacy and safety. A total of 51% of patients with refractory DLBCL treated with Yescarta^®^ (Gilead, Foster City, CA, USA) had a complete response [2], whereas Kymriah^®^ (Novartis, Bâle, Switzerland) provided a response in 52% and 81% of patients with DLBCL and acute lymphoblastic leukemia, respectively [3,4,5,6]. More recently, Breyanzi^®^ (Juno Therapeutics, Bristol-Myers Squibb, New-York, NY, USA) was shown to induce a complete response rate of 53% in the treatment of adult patients with relapsed or refractory (R/R) DLBCL [7,8].

The first step in the preparation of these drugs is the apheresis collection of the mononuclear cell (MNC), known as leukapheresis. This procedure allows the collection of a large enough number of T lymphocytes to initiate CAR-T cell culture. The collection of an adequate number of T lymphocytes is essential to ensure manufacturing success. [9,10].

Leukapheresis requires the use of several medical devices (MDs), such as catheters, tubing, filters, connectors, collection bags, and anticoagulant bags.

Most of these MDs are made of plasticized polyvinylchloride (PVC), and are therefore subject to content–container interactions with the patients’ blood, such as the release of the plasticizers contained in these MDs. Indeed, plasticizers such as di(2-ethylhexyl)-phthalate (DEHP) are added to PVC MDs to optimize their physical properties, but are not covalently bound, and can leach from PVC when the surface comes into contact with fluids [11]. Due to its operating conditions (long contact time, large contact area), leukapheresis is a medical procedure with a high risk of exposure to PVC plasticizers.

Few studies have been conducted on patients exposed to plasticizers during apheresis. Koch et al. assessed the urinary DEHP metabolites of six patients who underwent continuous thrombocytapheresis, and showed a DEHP exposure of 32.3 μg/kg/d [12], whereas Buchta et al. estimated it to be 7.2 μg/kg/d [13]. These exposure doses of DEHP are close to the tolerable daily intake (TDI) of 50 μg/kg/d established by the EFSA (European Food Safety Authority), and show that patients are significantly exposed during thrombocytapheresis [14]. Nevertheless, none of these works have focused on leukapheresis, or studied alternative plasticizers to DEHP.

Patient exposure via apheresis MDs remains problematic due to a potential risk of reprotoxicity of some plasticizers [15,16]. It has also been demonstrated that some of these chemicals are classified as endocrine disruptors (EDs) [17]. Indeed, recent studies show that contaminants such as phthalates, and particularly DEHP, disrupt several hormonal processes, such as steroid hormone and thyroid systems [16]. DEHP can disrupt thyroid hormone signaling by reducing the circulating thyroid hormone levels, thus affecting growth, development, and differentiation—especially in the developing brain [17,18]; it may also modify steroid hormone metabolism and balance by altering synthesis and/or breakdown of testosterone, follicle stimulation hormone, luteinizing hormone, or other hormones involved in gamete physiology, fertility, implantation, fetal morphogenesis, pregnancy outcomes, and post-birth diseases [19,20]. Although most of DEHP’s alternatives are still not classified by the ECHA concerning their ED capacity, due to insufficient or lacking data, some of the—such as DINP (diisononyl phthalate), DINCH (1,2-cyclohexane dicarboxylic acid diisononyl ester), and DEHA (bis(2-ethylhexyl) adipate)—exert ending points, such as the carcinogenic effects that were assessed in mice models [15].

It is therefore essential to identify and quantify the plasticizers released by the MD(s) in contact with the patient’s blood during a cytapheresis procedure.

The aim of our study was to evaluate the degree of exposure to plasticizers during leukapheresis for downstream CAR-T manufacturing, but also to evaluate the exposure of T cells. To this end, we developed a model simulating clinical conditions, thus reflecting the exposure of patients and T lymphocytes to these plasticizers via MDs.

## 2. Materials and Methods

### 2.1. Drugs and Medical Devices

The drugs and medical devices used in the model are summarized in Table 1.

To perform the model, we used a Spectra Optia^®^ Therumo BCT (N°1P01051)) apheresis device.

### 2.2. Reagents

The plasticizers used for analytical quantification were DEHP (CAS 117-81-7, product number 80030, batch BCBW8334), bis(2-ethylhexyl) adipate (DEHA) (CAS 103-23-1, product number 02137, batch BCBV4867), and benzyl butyl phthalate (BBP, used as internal standard) (CAS 85-68-7, product number 308501, batch MKBZ4553V), purchased from Sigma-Aldrich (St. Louis, MO, USA). The reagents used to prepare the simulant were absolute ethanol (Sigma-Aldrich, St. Louis, MO, USA) and sterile water (Versylene^®^, Fresenius, Bad Homburg, Germany).

### 2.3. Gas Chromatography–Mass Spectrometry (GC–MS)

The identification and quantification of plasticizers was performed with a Clarus 500 GC–MS chromatograph (PerkinElmer, Waltham, MA, USA) using a 70 eV electron impact ionization and an Optima 5 Accent, 5% diphenyl 95% dimethylpolysiloxane (30 m × 0.25 µm × 0.25 mm ID) capillary column (Macherey-Nagel, Düren, Germany). The mass detector was a simple quadrupole.

### 2.4. Methods

The study consisted of two parts: the first corresponded to the analysis of the plasticizers present in the MDs used for the apheresis procedure, and the second was dedicated to the assessment of the amounts of these plasticizers released from the MDs during a simulated procedure.

### 2.5. Analysis of Plasticizers in MDs

**Figure 1:**
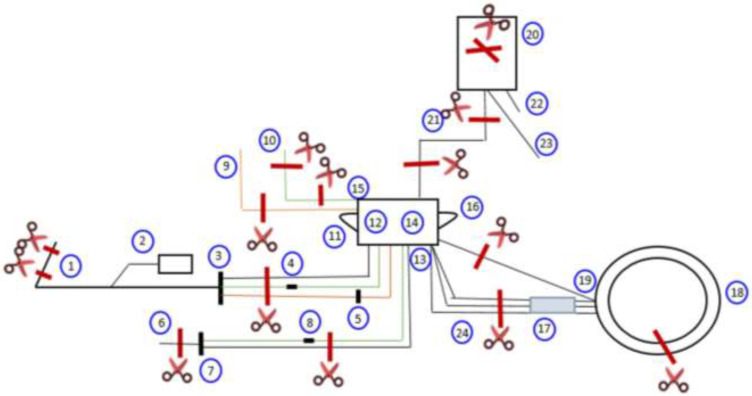
DEHP contents in the apheresis kit, and details of the sections for its determination. 1: Inlet line; 2: diversion bag; 3: inlet line manifold; 4: inlet saline line clamp (red); 5: anticoagulant (AC) check valve; 6: return line; 7: return line manifold; 8: return saline line clamp (blue); 9: AC line (orange); 10: saline line (green); 11: inlet line trap; 12: inlet pressure sensor diaphragm; 13: centrifuge pressure sensor diaphragm; 14: reservoir; 15: return pressure sensor diaphragm; 16: collect pressure sensor diaphragm; 17: centrifuge loop; 18: channel; 19: connector; 20: collection Bag; 21: collect line; 22: sample bulb assembly; 23: accessory line; 24: manifold to channel.

All MDs were divided into several sections that may contain different types and amounts of plasticizers (Figure 1). Each section was then analyzed as follows: The first step for the identification and quantification of plasticizers in MDs was the extraction of plasticizers from the MD matrix, performed according to the method published by Bourdeaux et al. [18]. We introduced a sample of 10 mg of the MDs into a 25 mL flask filled with chloroform containing the internal standard (BBP). After one hour of contact, the solution was homogenized, and 1 mL was taken and introduced into a glass chromatography vial for GC–MS analysis.

The analysis of plasticizers in the MDs was carried out by GC–MS [18]. In brief, the oven started at 200 °C for 1 min, and then was gradually raised to a temperature of 300 °C for 6 min and, finally, maintained at 300 °C for 7 min. The rate of helium N55 (mobile phase) in the column was 1.20 mL min^−1^. The total analysis time was 15 min (21 min between two injections). The injector temperature was set up at 300 °C. Specific mass-to-charge ratios (*m*/*z* values) were chosen per plasticizer—185; 217; 329 (for ATBC (acetyl tri-n-butyl citrate)), 149; 167; 279 (for DEHP), 112; 129; 241 (for DEHA), 112; 121; 261 (for DEHT (di(2-ethylhexyl)-phthalate)), 155; 299; 252 (for DINCH), 91; 149 (for BBP), 149; 293 (for DINP), and 193; 305; 323 (for TOTM (trioctyl trimellitate)) (the ions in bold were those used for the quantification)—in order to perform an analysis in SIR (single ion ratio) mode.

The LOD (limit of detection) and LOQ (limit of quantification) values for both plasticizers were 0.03 µg/mL and 0.25 µg/mL, respectively.

### 2.6. Analysis of Plasticizers in the Apheresis Model

#### Model

This model was used to estimate the total leaching of plasticizers from the PVC tubing during a leukapheresis procedure. Therefore, we used the continuous-flow mononuclear cell collection (CMNC) method on the Spectra Optia, which was used for pre-CAR-T-cell collection. The CMNC protocol allows the collection of mononuclear cells, including monocytes, lymphocytes, CD34+ cells, and dendritic cells. Biometric parameters of a “standard” simulated patient (male, 170 cm, 70 kg, and hematocrit 48%) were set up. The model conditions established according to the clinical practice were as follows:

- Simulant:
  - A 50/50 *v/v* mixture of absolute ethanol and water was chosen to simulate the blood due to its similar but higher capacity to extract DEHP from the PVC matrix than that of blood, along with the absence of the enzymes likely to metabolize DEHP [19];
  - The simulant volume was 5 L in order to correspond to the mean blood volume of a normal adult; it was contained in a closed 5 L glass flask;
- Flow rates were set up in accordance with the pre-CAR-T cells’ leukapheresis standard. Blood sampling rate was set up at 65 mL/min, and ACD-A flow rate at 1.1 mL/min;
- Contact time: The collection time was dependent on the patient. In the literature, the average collection time was 2–5 h [20]; therefore, the experiment was performed for a duration of 185 min, which is the average duration of a pre-CAR-T cell collection procedure performed in our hospital (*n* = 7).

**Figure 2:**
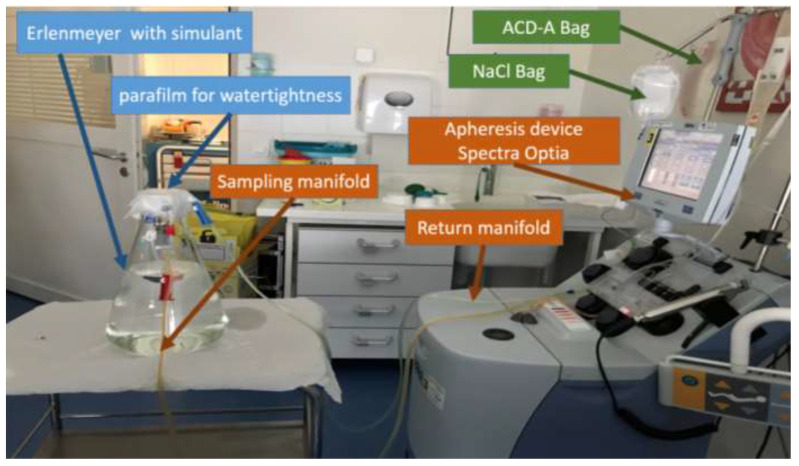
The apheresis model used in the study.

The modelling was performed in triplicate, from which three samples were collected (Figure 2). Briefly, the simulant was collected from the Erlenmeyer flask through the sampling manifold; it then went into a reservoir in the apheresis device, where it was mixed with ACD-A (an anticoagulant) and 0.9% saline solution (which were delivered to the reservoir through independent tubes). The simulant and both drugs then passed through a centrifugation ring, before returning to the Erlenmeyer flask through the return manifold. The Erlenmeyer flask was sealed with several layers of parafilm to prevent evaporation and, therefore, close the system.

### 2.7. Extraction of Plasticizers from the Model

Fifty-milliliter simulant samples were collected at the end of the experiment, i.e., after 185 min. After collection, they were stored in hemolysis tubes in a refrigerator between 4 and 8 °C until plasticizer quantification by GC–MS. A liquid–liquid extraction was then performed in GC–MS [18]. Briefly, for each analysis, 600 µL of the ethanolic solution was taken and added to 600 µL of a 2 µg/mL BBP–chloroform solution (internal standard). After homogenization (vortexing at 20 Hz for 30 s), the samples were centrifuged (3500 rpm, 5 min). Finally, the chloroform phase (the lower of the two phases) was taken for analysis.

### 2.8. Identification and Quantification of Released Plasticizers

The analysis of plasticizers in our model was carried out by GC–MS according to the method described above.

#### Expression of Results

The amount of each plasticizer in the MDs was expressed as a percentage of the mass (%), i.e., in grams per 100 g of PVC tubing. For the simulant, the chromatographic analysis gives a plasticizer concentration in milligrams per liter (mg/L). However, in order to evaluate the exposure of the patients to the plasticizers during leukapheresis, it was appropriate to express the results in mg/kg/day, with a considered volume of 5 L and a patient mass of 70 kg. Therefore, we calculated the exposure dose as follows:

Dp = (Cp × Vsimulant)/BW;
Cp = measured concentration of plasticizer (mg/L);
Dp = administered daily intake of plasticizer (mg/kg/d);
Vsimulant = volume of the simulant (5 L);
BW = body weight (70 kg).

## 3. Results

DEHP was found to be the main plasticizer in the apheresis set, whereas DEHA was detected at trace levels. We did not identify (<LOD) any other plasticizer that could be analyzed by the method of [18] (i.e., ATBC, DEHT, DINP, DINCH, TOTM). The DEHP content of each section of the MDs ranged from 25% to 59%. We also found DEHP in the ACD-A bag. Full results are presented in detail in Table 2. 

After 185 min, the average concentration of DEHP released in the ethanolic simulant was 19.78 ± 2.289 µg/mL. This concentration corresponds to a DEHP dose of 98.90 mg, i.e., an average exposure dose of 1.41 ± 0.16 mg/kg/day for a 70 kg patient. The results for each assay are detailed in Table 3.

## 4. Discussion

The strength of this study is the fact that it is the first to assess the exposure of patients to plasticizers during a pre-CAR-T-cell-apheresis procedure. We developed a model reflecting the clinical conditions of pre-CAR-T-cell apheresis using real-life parameters (especially apheresis devices used in inpatients, set up with usual human parameters). We showed that patients could be exposed to 98.90 ± 11.42 mg of DEHP during their treatment at the hospital. This corresponds, for a 70 kg adult, to an exposure dose of 1.41 mg/kg/day—almost 30–40 times higher than the ECHA recommended tolerable daily intake (TDI) of 0.036 mg/kg/day by oral administration [17]. This TDI is an estimate of the amount of a substance that can be taken daily over a lifetime without an appreciable health risk. Previous studies investigating the exposure to plasticizers during thrombocytapheresis found lower DEHP exposure doses, between 7.2 and 32 µg/kg/day [12,13]. Several reasons might explain the differences in our results. First, the modalities of the two procedures—leukapheresis and thrombocytapheresis—are not similar; the length of the procedure was higher in our case and, therefore, the contact time was also higher (185 min, while for Buchta et al. and Koch et al. the contact time was 60 min and 62 min, respectively). Then, the total amount of DEHP that patients were exposed to during apheresis was calculated from plasma or urinary samples of volunteers based on the level of DEHP or its metabolites, whereas we used an ex vivo model using an ethanolic simulant. We found a DEHP exposure dose ~45 times higher than in the literature, due to methodological differences, including the use of a different simulant, which could lead to a significant overestimation. The use of EtOH/H_2_O (50/50 *v*/*v*) as a simulant is the main limitation of our study, providing an overestimation of exposure doses. However, this ethanolic simulant is one of the synthetic simulants considered as being relevant for the evaluation of plasticizer migration into human blood products, according to Thelliez et al. [21], even if the simulant’s chemical composition does not match that of the labile blood products. Due to the major drawbacks of natural simulants such as porcine or bovine blood (potential contamination from environmental pollutants/phthalates and/or difficulties in extracting the analytes), water/ethanol mixture is among the most relevant synthetic simulants, thanks to its easy and safe use, low cost, ease of supply and disposal, and inter-batch reproducibility, unlike blood products. Moreover, in a previous work we have already developed a method for the extraction of plasticizers from EtOH/H_2_O simulant [1]). Luo et al. also showed that the ethanolic simulant has an extraction ability approximately 40 times higher than that of blood [19]. Once this factor taken into account, the exposure dose obtained from our study (19.78/40 = 0.50 µg/mL) would therefore be in the range of those in the literature.

However, despite a probable overestimation, the possible exposure to phthalates raises two problems: First, patients undergoing an apheresis procedure are here exposed to DEHP doses from which endocrine disruption and/or reprotoxic effects have been identified. Indeed, DEHP is associated with endocrine disruption effects on the reproductive and thyroid systems [22,23]. Despite its simulant-nature-based overestimation, the concentration of DEHP found in the simulant is in the range of those that may exhibit a transcriptional activity on androgen receptors [24], or can affect the production of testosterone [25]. Moreover, as DEHP is known to be greatly metabolized in vivo after oral exposure, this suggests a low probability of direct tissue exposure to the parent substance, to the benefit of its active metabolites, such as hydroxylated/oxidized metabolites, which induce even more pronounced effects in reporter genes [26]. In addition, DEHP has also been categorized as carcinogenic, mutagenic, or toxic for reproduction 1B under the Classification Labeling and Packaging (CLP) Regulation (Testai et al., 2016; Regulation (EU) 2017/745 [27]), due to suspected reprotoxicity in humans [10]. Moreover, DEHP—and especially its primary metabolite MEHP (mono(2-ethylhexyl)phthalate)—is also responsible for a significant decrease in cell viability as soon as reaching blood concentrations of 0.05 mg/mL and 0.01 mg/mL, respectively [28]. Our model (simulant) does not allow us to measure the exposure to MEHP, due to the absence of the esterase contained in the bloodstream (therefore, no metabolism can occur). In addition, it would have been biased to use blood as a simulant, because of the presence of DEHP as a plasticizer in the blood bags. [29]. According to Munch et al., the MEHP/DEHP ratio in blood after 3 h of an extracorporeal membrane oxygenation (ECMO) procedure is 2.7/97.3 [30], which leads us to estimate an MEHP blood concentration of 0.55 µg/mL—a dose below its cytotoxic concentration.

Nevertheless, during pre-CAR-T cells’ apheresis, the patient’s exposure is limited to a single or small number of procedures, which puts things in perspective concerning the risk to their health. In contrast, other clinical situations that require leukapheresis (e.g., mononuclear cell donations (for hematopoietic allogenic transplants), hematopoietic autologous transplantations, or extracorporeal photochemotherapy) may require multiple treatments and, therefore, several instances of exposure to phthalates

Therefore, this may be the most important issue of this exposure, resulting in a possible decrease in the CAR-T cells’ treatment quality. Indeed, phthalates have also been suggested as modulators of the immune system. Turner et al. showed that the exposure of lymphocytes to DEHP leads to a significant decrease in their numbers and an inhibition of the lymphocytes’ mitosis [31]. Moreover, Rosado-Berrios et al. studied the mitochondrial membrane permeability, reactive oxygen species (ROS) generation, and activation of caspases 3 and 7 in human lymphoblasts exposed to DEHP. They showed that the 24 h half-maximal inhibitory concentration (IC_50_) was ~250 µmol/L (97.5 µg/mL) for both MEHP and DEHP, and 80 and 100 µmol/L, respectively, at 72 h. Although the IC_50_ was not reached in our experiment, such concentrations can lead to a decrease in the efficiency and quality of the treatment—especially where lymphocytes are crucial [32]. Finally, various lymphocyte models were used to assess the effects of phthalates in T-cell populations, and DEHP was found to be an activator of inflammation [33,34], increasing the release of Th2-associated mediators from lymphocytes, with modulation of the Th1/Th2 balance [35,36,37,38], probably impacting their anti-inflammatory and immunomodulatory effects. Since the number and quality of lymphocytes collected from the patient are key and limiting factors in the preparation of CAR-T cells, aberrant activation of T cells or toxicity due to DEHP (such as modification of their anti-inflammatory and immunomodulatory effects) could decrease the manufacturing success of this cell therapy.

Finally, our work highlights the fact that DEHP is still present as a plasticizer in most parts of the MDs used for leukapheresis, despite the health concerns relative to its proven endocrine disruption effects, which have been taken into account by the European authorities, resulting in restrictions of its use given by the European Regulation 2017/745 (applicable in May 2021). However, in other clinical situations of high risk of exposure, such as extracorporeal circulation (ECMO) (Fernandez-Canal et al., 2018) or hemofiltration (Bernard et al., 2020), DEHP has been replaced in MDs by different alternatives, such as tris(2-ethylhexyl)trimellitate (TOTM) and bis(2-ethylhexyl) adipate (DEHA) [18,39]. In our case, the apheresis set contains almost exclusively DEHP, at an amount ranging from 25% to 59%. This may be explained by the fact that DEHP stabilizes red blood cells (RBCs), thus increasing the shelf life and storage of those cells [40]. The investigations to find secure alternative plasticizers for these MDs are still ongoing, and face the challenging issue of the stability of RBCs.

In conclusion, our work highlights how DEHP is released during leukapheresis. Although the consequences of this exposure to DEHP are minimal for patients requiring CAR-T cells, it can have a real impact on the quality of their treatment. It is therefore essential to find an alternative to DEHP.

## Figures and Tables

**Table 1:** Characteristics of drugs and medical devices used in the study.

Name	Supplier	Batch Number	Shelf-Life	Use
ACD-A	Fresenius Kabi	FC19H21L	2021/01	Anticoagulation
0.9% sodium chloride	B Braun Medical	15PDD680	03/22	Priming and restitution
Medical devices				
Spectra Optia^®^ IDL Set (N°10315)	Terumo BCT	1911273130	01/10/2021	Apheresis device

**Table 2:** Amounts of plasticizers in the different sections of the apheresis set, and in the anticoagulant bag.

Location of Cut	Detail Cut	Average Content of Plasticizer (% i.e., g/100 g of PVC) (Mean ± SD)	Relative Standard Deviation (RSD)
Inlet line (1)	Section 1	DEHP ^1^ = 36.89 ± 2.03	5.5%
	Section 2	DEHP ^1^ = 34.43 ± 2.81	8.2%
	Section 3	NA ^2^	NA ^2^
Inlet line manifold (3)	Orange	DEHP ^1^ = 39.45 ± 1.94 DEHA ^2^ < LOQ	4.9%
	White	DEHP ^1^ = 40.55 ± 3.61	8.9%
	Green	DEHP ^1^ = 49.55 ± 1.30	2.6%
Return line (6)		DEHP ^1^ = 30.69 ± 1.73 DEHA ^2^ < LOQ	5.6%
Manifold to channel (24)	White 1	DEHP ^1^ = 29.06 ± 0.53	1.8%
	White 2	DEHP ^1^ = 28.69 ± 1.12 DEHA < LOQ	3.9%
	Pink	DEHP ^1^ = 31.99 ± 1.56 DEHA ^2^ < LOQ	4.9%
	Yellow	DEHP ^1^ = 27.45 ± 1.72 DEHA^2^ < LOQ	6.3%
Return line manifold (7)	White	DEHP ^1^ = 30.56 ±1.66	5.4%
	Green	DEHP ^1^ = 31.13 ± 0.48	1.5%
Channel (18)		NA ^3^	NA ^3^
Anticoagulant (AC) line (9)		DEHP ^1^ = 32.45 ± 1.26 DEHA ^2^ < LOQ	3.9%
Saline line (10)	Section 1	DEHP ^1^ = 59.44 ± 5.69	9.6%
	Section 2	DEHP ^1^ = 34.86 ± 1.56	4.5%
Collect line (21)		DEHP ^1^ = 24.99 ± 0.97 DEHA ^2^ < LOQ	3.9%
Collection bag (20)	Pocket	NA ^3^	NA ^3^
	Manifold	DEHP ^1^ = 28.34 ± 2.16 DEHA < LOQ	7.6%
ACD-A Bag	Spike port	DEHP ^1^ = 45.42 ± 20.17	44.4%
	Pocket	DEHP ^1^ = 28.29 ± 1.69	6.0%

^1^ DEHP: bis(2-ethylhexyl) phthalate; ^2^ DEHA: bis(2-ethylhexyl) adipate; ^3^ NA: not applicable.

**Table 3:** Concentrations of DEHP released during our apheresis model with water/ethanol simulant.

Assay Number	C_DEHP_^1^ in Each Sample (µg/mL)	Mean C_DEHP_^1^ ± SD (µg/mL)	RSD	Calculated Dose (mg/kg/day)
1	17.16	17.1 ± 0.70	4.1%	1.2
	17.82			
	16.42			
2	19.86	19.9 ±0.09	0.4%	1.4
	20			
	19.84			
3	22.28	22.3 ±0.52	2.3%	1.6
	21.8			
	22.84			

^1^ C_DEHP_: concentration of bis(2-ethylhexyl) phthalate in the simulant.

## Data Availability

The data presented in this study are available in supplementary material.

## Supplementary Materials

The following are available online at https://www.mdpi.com/article/10.3390/toxics10020079/s1, Table S1: Raw data of the quantification of plasticizer in the different sections of the apheresis set, and in the anticoagulant bag. Table S2: Raw data of the quantification of DEHP released in the water/ethanol.

## References

[1] Fitzmaurice C., Akinyemiju T.F., Al Lami F.H., Alam T., Alizadeh-Navaei R., Allen C., Alsharif U., Alvis-Guzman N., Amini E., Global Burden of Disease Cancer Collaboration (2018). Global, Regional, and National Cancer Incidence, Mortality, Years of Life Lost, Years Lived with Disability, and Disability-Adjusted Life-Years for 29 Cancer Groups, 1990 to 2016: A Systematic Analysis for the Global Burden of Disease Study. JAMA Oncol..

[2] Neelapu S.S., Locke F.L., Bartlett N.L., Lekakis L.J., Miklos D.B., Jacobson C.A., Braunschweig I., Oluwole O.O., Siddiqi T., Lin Y. (2017). Axicabtagene Ciloleucel CAR T-Cell Therapy in Refractory Large B-Cell Lymphoma. N. Engl. J. Med..

[3] Schuster S.J., Svoboda J., Chong E.A., Nasta S.D., Mato A.R., Anak Ö., Brogdon J.L., Pruteanu-Malinici I., Bhoj V., Landsburg D. (2017). Chimeric Antigen Receptor T Cells in Refractory B-Cell Lymphomas. N. Engl. J. Med..

[4] Schuster S.J., Bishop M.R., Tam C.S., Waller E.K., Borchmann P., McGuirk J.P., Jäger U., Jaglowski S., Andreadis C., Westin J.R. (2019). Tisagenlecleucel in Adult Relapsed or Refractory Diffuse Large B-Cell Lymphoma. N. Engl. J. Med..

[5] Meng J., Wu X., Sun Z., Xun R., Liu M., Hu R., Huang J. (2021). Efficacy and Safety of CAR-T Cell Products Axicabtagene Ciloleucel, Tisagenlecleucel, and Lisocabtagene Maraleucel for the Treatment of Hematologic Malignancies: A Systematic Review and Meta-Analysis. Front. Oncol..

[6] Westin J.R., Kersten M.J., Salles G., Abramson J.S., Schuster S.J., Locke F.L., Andreadis C. (2021). Efficacy and Safety of CD19-Directed CAR-T Cell Therapies in Patients with Relapsed/Refractory Aggressive B-Cell Lymphomas: Observations from the JULIET, ZUMA-1, and TRANSCEND Trials. Am. J. Hematol..

[7] Food and Drug Administration (2021). FDA Approves Lisocabtagene Maraleucel for Relapsed or Refractory Large B-Cell Lymphoma. https://www.fda.gov/drugs/resources-information-approved-drugs/fda-approves-lisocabtagene-maraleucel-relapsed-or-refractory-large-b-cell-lymphoma.

[8] Abramson J.S., Palomba M.L., Gordon L.I., Lunning M.A., Wang M., Arnason J., Mehta A., Purev E., Maloney D.G., Andreadis C. (2020). Lisocabtagene Maraleucel for Patients with Relapsed or Refractory Large B-Cell Lymphomas (TRANSCEND NHL 001): A Multicentre Seamless Design Study. Lancet.

[9] Fesnak A., Lin C., Siegel D.L., Maus M.V. (2016). CAR-T Cell Therapies from the Transfusion Medicine Perspective. Transfus. Med. Rev..

[10] Tyagarajan S., Schmitt D., Acker C., Rutjens E. (2019). Autologous Cryopreserved Leukapheresis Cellular Material for Chimeric Antigen Receptor–T Cell Manufacture. Cytotherapy.

[11] Hanawa T., Muramatsu E., Asakawa K., Suzuki M., Tanaka M., Kawano K., Seki T., Juni K., Nakajima S. (2000). Investigation of the Release Behavior of Diethylhexyl Phthalate from the Polyvinyl-Chloride Tubing for Intravenous Administration. Int. J. Pharm..

[12] Koch H.M., Bolt H.M., Preuss R., Eckstein R., Weisbach V., Angerer J. (2005). Intravenous Exposure to Di(2-Ethylhexyl)Phthalate (DEHP): Metabolites of DEHP in Urine after a Voluntary Platelet Donation. Arch. Toxicol..

[13] Buchta C., Bittner C., Höcker P., Macher M., Schmid R., Seger C., Dettke M. (2003). Donor Exposure to the Plasticizer Di(2-Ethylhexyl)Phthalate during Plateletpheresis. Transfusion.

[14] European Chemicals Agency Recommendation of the European Chemicals Agency of 10 July 2019 to Amend the Annex XIV Entries to REACH of DEHP, BBP, DBP and DIBP. https://echa.europa.eu/documents/10162/13640/axiv_amend_recommendation_phthalates_july2019_en.pdf/1889866a-bec3-fe16-6322-67c16a13b09d.

[15] Testai E., Hartemann P., Rastogi S.C., Bernauer U., Piersma A., De Jong W., Gulliksson H., Sharpe R., Schubert D., Rodríguez-Farre E. (2016). The Safety of Medical Devices Containing DEHP Plasticized PVC or Other Plasticizers on Neonates and Other Groups Possibly at Risk (2015 Update). Regul. Toxicol. Pharmacol..

[16] The Danish Environmental Protection Agency (2014). Alternatives to Classified Phthalates in Medical Devices.

[17] Bis(2-ethylhexyl) Phthalate—Substance Information—ECHA. https://echa.europa.eu/fr/substance-information/-/substanceinfo/100.003.829.

[18] Bourdeaux D., Yessaad M., Chennell P., Larbre V., Eljezi T., Bernard L., Sautou V. (2016). Analysis of PVC Plasticizers in Medical Devices and Infused Solutions by GC–MS. J. Pharm. Biomed. Anal..

[19] Luo H., Sun G., Shi Y., Shen Y., Xu K. (2014). Evaluation of the Di(2-Ethylhexyl)Phthalate Released from Polyvinyl Chloride Medical Devices That Contact Blood. SpringerPlus.

[20] Korell F., Laier S., Sauer S., Veelken K., Hennemann H., Schubert M.-L., Sauer T., Pavel P., Mueller-Tidow C., Dreger P. (2020). Current Challenges in Providing Good Leukapheresis Products for Manufacturing of CAR-T Cells for Patients with Relapsed/Refractory NHL or ALL. Cells.

[21] Thelliez A., Hénard G., Delorme B., Chatellier S., Danel C., Ducoroy L., Dupont A., Garrigue D., Genay S., Goossens J.-F. (2021). Specification and Evaluation of Plasticizer Migration Simulants for Human Blood Products: A Delphi Study. Biomolecules.

[22] Andra S.S., Makris K.C. (2012). Thyroid Disrupting Chemicals in Plastic Additives and Thyroid Health. J. Environ. Sci. Health Part C.

[23] Huang P.-C., Chang W.-H., Wu M.-T., Chen M.-L., Wang I.-J., Shih S.-F., Hsiung C.A., Liao K.-W. (2020). Characterization of Phthalate Exposure in Relation to Serum Thyroid and Growth Hormones, and Estimated Daily Intake Levels in Children Exposed to Phthalate-Tainted Products: A Longitudinal Cohort Study. Environ. Pollut..

[24] Engel A., Buhrke T., Imber F., Jessel S., Seidel A., Völkel W., Lampen A. (2017). Agonistic and Antagonistic Effects of Phthalates and Their Urinary Metabolites on the Steroid Hormone Receptors ERα, ERβ, and AR. Toxicol. Lett..

[25] Mankidy R., Wiseman S., Ma H., Giesy J.P. (2013). Biological Impact of Phthalates. Toxicol. Lett..

[26] Kambia N.K., Séverin I., Farce A., Moreau E., Dahbi L., Duval C., Dine T., Sautou V., Chagnon M.-C. (2019). In Vitro and in Silico Hormonal Activity Studies of Di-(2-Ethylhexyl)Terephthalate, a Di-(2-Ethylhexyl)Phthalate Substitute Used in Medical Devices, and Its Metabolites. J. Appl. Toxicol. JAT.

[27] (2017). Règlement (UE) 2017/745 du Parlement Européen et du Conseil. https://eur-lex.europa.eu/legal-content/FR/TXT/PDF/?uri=CELEX:32017R0745.

[28] Eljezi T., Pinta P., Nativel F., Richard D., Pinguet J., Roy O., Sautou V., Grimandi G., Moreau E. (2019). In Vitro Cytotoxic Effects of Secondary Metabolites of DEHP and Its Alternative Plasticizers DINCH and DINP on a L929 Cell Line. Int. J. Hyg. Environ. Health.

[29] Wittassek M., Wiesmüller G.A., Koch H.M., Eckard R., Dobler L., Müller J., Angerer J., Schlüter C. (2007). Internal Phthalate Exposure over the Last Two Decades—A Retrospective Human Biomonitoring Study. Int. J. Hyg. Environ. Health.

[30] Fernandez-Canal C., Pinta P.-G., Eljezi T., Larbre V., Kauffmann S., Camilleri L., Cosserant B., Bernard L., Pereira B., Constantin J.-M. (2018). Patients’ Exposure to PVC Plasticizers from ECMO Circuits. Expert Rev. Med. Devices.

[31] Turner J.H., Petricciani J.C., Crouch M.L., Wenger S. (1974). An Evaluation of the Effects of Diethylhexyl Phthalate (DEHP) on Mitotically Capable Cells in Blood Packs. Transfusion.

[32] Rosado-Berrios C.A., Vélez C., Zayas B. (2011). Mitochondrial Permeability and Toxicity of Diethylhexyl and Monoethylhexyl Phthalates on TK6 Human Lymphoblasts Cells. Toxicol. In Vitro.

[33] Nygaard U.C., Ulriksen E.S., Hjertholm H., Sonnet F., Bølling A.K., Andreassen M., Husøy T., Dirven H. (2021). Immune Cell Profiles Associated with Measured Exposure to Phthalates in the Norwegian EuroMix Biomonitoring Study—A Mass Cytometry Approach in Toxicology. Environ. Int..

[34] Han Y., Wang X., Pang X., Hu M., Lu Y., Qu J., Chen G. (2019). Di-(2-Ethylhexyl)-Phthalate Interferes with T-Follicular Helper Cell Differentiation and Cytokine Secretion through Signaling Lymphocytic Activation Molecule Family Member-1. J. Immunotoxicol..

[35] Yamashita U., Sugiura T., Kuroda E. (2002). Effect of Endocrine Disrupters on Immune Responses In Vitro. J. UOEH.

[36] Yamashita U., Sugiura T., Yoshida Y., Kuroda E. (2003). Effect of Endocrine Disrupters on Thymocytes In Vitro. J. UOEH.

[37] Oh P.-S., Lim K.-T. (2009). Suppressive Effect of CTB Glycoprotein (75 KDa) on IL-4 Expression in Primary-Cultured Lymphocytes Treated with Di(2-Ethylhexyl) Phthalate. Naunyn. Schmiedebergs Arch. Pharmacol..

[38] Pei X., Duan Z., Ma M., Zhang Y., Guo L. (2014). Role of Ca/CaN/NFAT Signaling in IL-4 Expression by Splenic Lymphocytes Exposed to Phthalate (2-Ethylhexyl) Ester in Spleen Lymphocytes. Mol. Biol. Rep..

[39] Gimeno P., Thomas S., Bousquet C., Maggio A.-F., Civade C., Brenier C., Bonnet P.-A. (2014). Identification and Quantification of 14 Phthalates and 5 Non-Phthalate Plasticizers in PVC Medical Devices by GC-MS. J. Chromatogr. B Analyt. Technol. Biomed. Life Sci..

[40] Rock G., Tocchi M., Ganz P.R., Tackaberry E.S. (1984). Incorporation of Plasticizer into Red Cells during Storage. Transfusion.

