# Supplementary material for: Ex Vivo Model to Assess the Exposure of Patients to Plasticizers from Medical Devices during Pre-CAR-T Cells’ Apheresis
Source: Toxics. 2022 Feb 8;10(2):79. doi: 10.3390/toxics10020079 (PMC8875078; doi:10.3390/toxics10020079)
Supplement: Supplementary file 1 [file toxics-10-00079-s001.zip › toxics-1544981-supplementary.pdf]

toxics

MDPI

Supplementary Materials: Ex Vivo Model to Assess the Exposure of Patients to Plasticizers from Medical Devices During Pre-CAR-T Cells' Apheresis

Raphaëlle Lautraite , Lise Bernard, Pascale Halle, Philip Chennell, Yoann Le Basle, Justyna Kanold and Valérie Sautou

Table S1. Raw data of the quantification of plasticizer in the different sections of the apheresis set, and in the anticoagulant bag.

MD	location of cut	detail cut	sample mass for screen	Plasticizer identification	Plasticizer in technical file	sample mass for identification	Major plasticizer Concentrations (µg/mL)	Major plasticizer Concentrations Mean (µg/mL)	SD	CV (%)	Major plasticizer content (g/100g)	Major plasticizer content Mean (g/100g)	SD	CV (%)	Minor plasticizer concentration (µg/mL)
Apheresis set	Inlet line (1)	section 1	10.4	DEHP	DEHP	11	165	156.1666667	15.2997821	9.80 %	37.5	36.89213396	2.03255597	5.50 %	/
						10.7	165				38.55140187				
						10	138.5				34.625				
		section 2	10.8	DEHP	DEHP	10.9	164	149.6666667	12.4230968	8.30 %	37.6146789	34.43287371	2.81370783	8.20 %	/
						11	142				32.27272727				
						10.7	143				33.41121495				
		section 3	10.5	DEHP +BBP	DEHP	NA									
			10	DEHP +BBP											
	Inlet line manifold (3)	Orange	10.7	DEHP + DEHA min	DEHP	10.9	168.8	165.6	2.8	1.70 %	38.71559633	39.44554568	1.93641287	4.90 %	< LOQ
						10.4	163.6				39.32692308				< LOQ
						10.2	164.4				40.29411765				< LOQ
		white	10.9	DEHP	DEHP	11	160.6	173.3333333	11.9102197	6.90 %	36.5	40.55256065	3.61453893	8.90 %	/
						10.5	175.2				41.71428571				
						10.6	184.2				43.44339623				
		green	10.7	DEHP + DEHA min	DEHP	10.5	214	212.6666667	1.52752523	0.70 %	50.95238095	49.55081064	1.29645947	2.60 %	/
						10.9	211				48.39449541				
						10.8	213				49.30555556				
	Return line (6)		10.5	DEHP +DEHA (min)	DEHP	10.4	136	128.0666667	8.00083329	6.20 %	32.69230769	30.69040131	1.73375452	5.60 %	< LOQ
						10.1	120				29.7029703				< LOQ

					10.8	128.2				29.67592593				< LOQ
Manifold to channel (24)	white 1	10.4	DEHP	DEHP	10.3	122	121.75	2.1015867	1.70%	29.61165049	29.0591907	0.52541719	1.80%	/
					10.5	119.5				28.45238095				
					10.6	124.5				29.36320755				
	white 2	10.9	DEHP + DEHA (min)	DEHP	10.9	121.2	122.4	4.13279566	3.40%	27.79816514	28.69477622	1.12188506	3.90%	< LOQ
					10.6	127				29.95283019				< LOQ
					10.5	119				28.33333333				< LOQ
	pink	10.8	DEHP + DEHA (min)	DEHP	10.8	139.6	136.1333333	8.92038863	6.60%	32.31481481	31.98925411	1.56364124	4.90%	< LOQ
					10.4	126				30.28846154				< LOQ
					10.7	142.8				33.36448598				< LOQ
	yellow	10.2	DEHP + DEHA (min)	DEHP	10.5	108.2	119.0666667	10.2256214	8.60%	25.76190476	27.45093795	1.72222853	6.30%	< LOQ
					11	120.5				27.38636364				< LOQ
					11	128.5				29.20454545				< LOQ
Return line manifold (8)	white	10	DEHP	DEHP	11	127	130.3333333	7.57187779	5.80%	28.86363636	30.55893906	1.65753298	5.40%	/
					10.2	125				30.6372549				
					10.8	139				32.17592593				
	green	10.2	DEHP	DEHP	10.6	130.2	131.1333333	1.44683563	1.10%	30.70754717	31.12869	0.47945944	1.50%	/
					10.3	130.4				31.65048544				
					10.7	132.8				31.02803738				
Channel (18)		10.9	DEHP +BBP	NA										
		10.5	DEHP +BBP											
Anticoagulant (AC) line (9)		10.2	DEHP + DEHA (min)	DEHP	10	124.4	134.2666667	12.1232559	9.00%	31.1	32.4469697	1.25780484	3.90%	< LOQ
					10	130.6				32.65				< LOQ
					11	147.8				33.59090909				< LOQ
Saline line (10)	section 1	10.6	DEHP	DEHP	10.5	243	245.8333333	26.3644331	10.70%	57.85714286	59.43510182	5.6877177	9.60%	/
					10.4	273.5				65.74519231				
					10.1	221				54.7029703				
	section 2	11	DEHP + DEHA (min)	DEHP	10.1	145	144.3333333	1.60727513	1.10%	35.89108911	34.86142364	1.55868151	4.50%	/
					10	142.5				35.625				

						11	145.5				33.06818182				
	collect line (25)		10.6	DEHP + DEHA (min)	DEHP	10.3	106.2	103	6.42806347	6.20%	25.77669903	24.98657263	0.97283611	3.90%	< LOQ
						10	95.6				23.9				< LOQ
						10.6	107.2				25.28301887				< LOQ
	Colection bag (24)	Poche	11	?	BTHC	/	/	/	/	/	/	/	/	/	/
						/	/								
						/	/								
		Tubulue	10.7	DEHP + DEHA (min)	?	11	134.5	122.8333333	10.40833	8.50%	30.56818182	28.33787066	2.15747817	7.60%	< LOQ
						10.9	114.5				26.26146789				< LOQ
						10.6	119.5				28.18396226				< LOQ
ACDA bag	Spike port	/	10.8	DEHP	DEHP ?	10.4	200.5	189.875	82.5775609	43.50%	48.19711538	45.42396656	20.1714897	44.40%	/
						10.3	206				50				
						10.3	112				27.18446602				
						10.9	300				68.80733945				/
						10.9	128				29.35779817				
						10	315				78.75				
						10.5	162				38.57142857				/
						10.6	95.5				22.52358491				
	Pocket	/	10.8	DEHP	DEHP	10.6	126	120	7.93725393	6.60%	29.71698113	28.29462344	1.68850765	6.00%	/
						10.7	123				28.73831776				
						10.5	111				26.42857143				

Table S2. Raw data of the quantification of DEHP released in the water/ethanol.

Simulation Solution	Simulation Number	DEHP Concentration (µg/mL)	DEHP Concentration Mean (µg/mL)	SD	CV	DEHP dose (mg)	Exposure Dose (mg/kg/jour)
Water/ethanol	Simulation 1	17.16	17.13	0.7	4.09%	85.67	1.22
		17.82					
		16.42					
	Simulation 2	19.86	19.9	0.09	0.44%	99.5	1.42
		20					
		19.84					
	Simulation 3	22.28	22.31	0.52	2.33%	111.53	1.59
		21.8					
		22.84					

Concentration (µg/mL) Mean	SD
19.78	2.588753454
Dose (mg) Mean	SD
98.9	12.94376727
Exposure dose Mean	SD
1.41	0.184910961
